# CD-Based Indices for Link Prediction in Complex Network

**DOI:** 10.1371/journal.pone.0146727

**Published:** 2016-01-11

**Authors:** Tao Wang, Hongjue Wang, Xiaoxia Wang

**Affiliations:** 1 School of Mathematics and Physics, North China Electric Power University, Baoding, Hebei Province, China; 2 School of Control and Computer Engineering, North China Electric Power University, Baoding, Hebei Province, China; Semmelweis University, HUNGARY

## Abstract

Lots of similarity-based algorithms have been designed to deal with the problem of link prediction in the past decade. In order to improve prediction accuracy, a novel cosine similarity index CD based on distance between nodes and cosine value between vectors is proposed in this paper. Firstly, node coordinate matrix can be obtained by node distances which are different from distance matrix and row vectors of the matrix are regarded as coordinates of nodes. Then, cosine value between node coordinates is used as their similarity index. A local community density index LD is also proposed. Then, a series of CD-based indices include CD-LD-k, CD*LD-k, CD-k and CDI are presented and applied in ten real networks. Experimental results demonstrate the effectiveness of CD-based indices. The effects of network clustering coefficient and assortative coefficient on prediction accuracy of indices are analyzed. CD-LD-k and CD*LD-k can improve prediction accuracy without considering the assortative coefficient of network is negative or positive. According to analysis of relative precision of each method on each network, CD-LD-k and CD*LD-k indices have excellent average performance and robustness. CD and CD-k indices perform better on positive assortative networks than on negative assortative networks. For negative assortative networks, we improve and refine CD index, referred as CDI index, combining the advantages of CD index and evolutionary mechanism of the network model BA. Experimental results reveal that CDI index can increase prediction accuracy of CD on negative assortative networks.

## Introduction

In our real world, many complex systems including social, biological, information and technology can be well described by networks where nodes represent individuals or agents, and links denote relations or interactions between nodes. In some networks, such as in protein-protein interaction networks [1,2], electrical power grid [3] and air transportation networks [4], how can we find out which pair of entities likely generate new links in the near future? These questions can be formed into the problem of link prediction, which attempts to estimate the likelihood of the existence of a link between two nodes, based on observed links or the attributes of nodes [3,5–7]. Instead of blindly checking all possible interactions, link prediction can sharply reduce the experimental costs if the predictions are accurate enough. In protein-protein interaction networks of biological systems, predicting possible interactions between proteins can help us to predict non-experimentally-observed interactions using the network of the known interactions for a certain organism [3,8,9]. Besides, in social networks, link prediction can be used for predicting potential consumers in on-line shopping network [10] and predicting potential friends for people based on current connections in on-line friendship networks [11]. In addition, in order to prevent social crimes or terrorist activities, link prediction also can be used to mine hidden connections between criminals [5].These above mentioned are social positive connections, while negative links, such as distrust links and foe links, also have important significance in our society and some meaningful researches have been done successfully[12,13]. Positive connections are concerned in this paper. More applications of link prediction, please reference to [5,6,14].

In recent years, lots of link prediction algorithms have been proposed. These algorithms can be broadly classified into three categories: node similarity-based algorithms [3,15,16], maximum likelihood algorithms [17] and probabilistic models [18,19]. Among them, similarity-based algorithms are the most intuitionistic and popular. Main assumption of this method is that the greater similarity indices are, the greater connection possibilities are. Prediction accuracy is a critical factor for measuring quality of a similarity index, so researchers have successfully proposed many methods based on similarity indices including neighbor-based methods [6,14]and distance-based methods [14,20]. Neighbor-based methods are based on the idea that two nodes are more likely to generate a link in the near future if they have more common neighbors, such as Salton [14], Sorensen [5], LHN [15], CN[21] etc. Low computational complexity is the greatest strength of these methods. For many networks with high clustering coefficient, neighbor-based methods can obtain satisfactory prediction accuracy. However, in some sparse networks with low clustering coefficient, it is difficult for neighbor-based methods to achieve high prediction accuracy [16,22,23]. This may be because such neighbor-based indices have underestimate and cannot calculate similarity between nodes without common neighbors [24]. Distance-based methods suppose that link probability is determined by distance or number of the shortest path between nodes, such as LP [20], Katz [14]and LHN-II [15]. Some of these methods can successfully resolve the weaknesses of lower prediction accuracy in low clustering coefficient networks [14]. However, some distance-based similarity indices are sensitive to the proportion of observed edges [21]. It means that their prediction accuracies will reduce obviously if the proportion of observed edges decreases in algorithm training set. As a matter of fact, under the effect of small world phenomenon [25], most of distances between nodes are equal and very small. So, distance-based algorithms sometimes do not work well [26–28]. In addition, most of distance-based similarity indices such as Katz and LHN-II are based on global information of network and have high time complexity as O(*n*^3^). Note that, there is a latent assumption that neighbor-based indices can be regarded as distance-based indices when the shortest path as 2 between nodes is only taken into account.

Besides, some indices based on node degrees are also proposed, such as PA [29] and RA [16] indices, and they can obtain satisfactory prediction accuracy on some networks. In 2013, in view of local communities, Carlo Vittorio Cannistraci proposed a series of LCP-based (Local-community-paradigm-based) indices including CAR, CPA, CAA, CRA and CJC which are proven as particularly effective algorithms by plenty of experiments [3]. While some of these algorithms are less robust [30], i.e., some algorithms can perform satisfactorily on a part of networks with specific properties but not universal methods. For example, PA index performs better on negative assortative coefficient networks but worse on positive assortative coefficient networks [16]. We will show you in the following that RA and LCP-based indices perform far from satisfactory on low clustering coefficient network. Actually, an evolving network model corresponds to a link prediction method [6,24,30–32]. PA index is the evolution mechanism of BA model [29] and BA networks are negative assortative [33], so such networks with negative assortative coefficient can be modeled as BA networks and the link prediction results of PA index on these networks are usually satisfactory. Fenhua Li [4] expanded PA index and analyzed social networks with it. In addition, Cui Ai-Xiang [34] studied an evolving model based on evolutionary mechanism CN index. More link prediction algorithms please reference to [6,26,35,36].

There are three big challenges in link prediction: prediction accuracy [6], complexity [14] and robustness [30]. In this paper, a series of distance-based similarity indices and a modified index are proposed for link prediction in order to overcome these problems mentioned above. Moreover, for improving the complexity of traditional shortest path algorithms, we propose a new method to calculate all shortest paths of un-weighted and undirected connected networks with time complexity as O(*n*^2^) at most, where *n* is the number of network nodes. All proposed similarity indices and improved index are termed as CD-based including CD-k, CD-LD-k, CD*LD-k and CDI for short. After compared with fifteen famous traditional similarity indices, experimental results on some real-world networks demonstrate the feasibility and effectiveness of the proposed CD-based similarity indices. CD-LD-k and CD*LD-k can effectively improve prediction accuracy, and CD-k index performs better on positive assortative coefficient networks. For negative assortative coefficient networks, improved CDI index can improve prediction accuracy of CD and PA index.

## Materials and Methods

### Definition

Let *G* = (*V*, *E*) be an un-weighted and undirected connected network, where *V* is a set of nodes in network *G*, number of nodes is |*V*| = *n*, *E* is a set of links(or edges), and number of links is |*E*| = *m*. Self-connection and multiple links are un-allowed. Connection of the network *G* can be represented as an adjacency matrix *A*, and its element *a*_*i*,*j*_ is 1 when a link between nodes *v*_*i*_ and *v*_*j*_ exists and 0 otherwise. If there is a link between nodes *v*_*i*_ and *v*_*j*_, one of the two nodes is a neighbor of the other one. In matrix *A*, sum of elements in line *i* is the degree of node *v*_*i*_ and denoted as *k*_*i*_. As a matter of fact, *k*_*i*_ is the number of neighbors of node *v*_*i*_. The similarity index of nodes *v*_*i*_ and *v*_*j*_ is defined as *s*_*i*,*j*_ which is supposed to be symmetry in undirected network, that is, *s*_*i*,*j*_ = *s*_*j*,*i*_. The higher the similarity index *s*_*i*,*j*_ is, the more likely the link between nodes *v*_*i*_ and *v*_*j*_ exists, so *s*_*i*,*j*_ is regarded as score of link between nodes *v*_*i*_ and *v*_*j*_.

#### Cosine Distance Index (CD)

For overcoming weakness of underestimate [24], a novel Cosine Distance Index (CD) based on distance between nodes is proposed in this paper. Firstly, for an un-weighted and undirected connected network *G*, *k*-distance matrix *L* is defined as

$$l_{i,j}=\{\begin{array}{cc} d_{i,j} & d_{i,j}\leq k\\ \infty & d_{i,j}>k \end{array}$$ (1)

where *d*_*i*,*j*_ is the shortest path between nodes *v*_*i*_ ∈ *V* and *v*_*j*_ ∈ *V*. Free threshold value *k* is a positive integer and *k* ∈ [1, *d*_max_], where *d*_max_ is the diameter of *G*, i.e., the maximum value of all shortest paths of *G*. It is clearly that *L* is equal to distance matrix of network *G* when *k* = *d*_max_. In the following, we set *k* = *d*_max_ unless give a special instruction.

Then network node coordinate matrix *C* is defined and its element

$$c_{i,j}=\{\begin{array}{c} \frac{1}{l_{i,j}},(i\neq j)\\ 1,(i=j) \end{array}$$ (2)

It is clearly that *c*_*i*,*j*_ = *c*_*j*,*i*_ and *C* = *A* + *I* for *k* = 1, where *I* is an identity matrix. Also, *C* = (*L* + *I*)^*α^ and *α* = −1, where D^*α^ is the *α*th entrywise power[37,38] of *D*. Actually, *c*_*i*,*j*_ can be regarded as similarity of nodes *v*_*i*_ and *v*_*j*_. The higher the value of *c*_*i*,*j*_, the higher the similarity of nodes *v*_*i*_ and *v*_*j*_. In order to analyze transportation networks, Liu H K [31] used inverse of geographical distance as the similarity index between two cities. But using *c*_*i*,*j*_ as node similarity, there will be vast number of identical similarities and this is not compatible with reality in complex networks.

So Cosine Distance Index (CD) is defined as

$$s_{i,j}^{CD}=\frac{\left(C_{i},C_{j}\right)}{\left\|C_{i}\right\|\times \left\|C_{j}\right\|}$$ (3)

where *C*_*i*_ is the *i*th row vector of coordinate matrix *C* and also denotes coordinate of node *v*_*i*_ in a *n*-dimensional Euclidean space, ‖*C*_*i*_‖ is module of *C*_*i*_. It can be found clearly that the higher the value of $s_{i,j}^{CD}$, the smaller the angle between vectors *C*_*i*_ and *C*_*j*_, and the higher the similarity measure between *v*_*i*_ and *v*_*j*_, so *C*_*i*_ is close to parallel with *C*_*j*_. Any two nodes have limited shortest path in a connected network, and there is non-zero similarity measure between them, namely, CD index of any two nodes can be calculated in a connected network and $s_{i,j}^{CD}\in \left[0,1\right]$, so CD can conquer underestimate problem of other algorithms obviously. In a realistic network, using CD index, it is difficult to get identical similarities for different pair of nodes. Note that (*v*_*i*_, *v*_*j*_) and (*v*_*j*_, *v*_*i*_) are the same pair of nodes, where *v*_*i*_ ≠ *v*_*j*_. Actually, for node pairs (*v*_*i*_, *v*_*j*_) and (*v*_*h*_, *v*_*j*_) where *v*_*i*_ ≠ *v*_*j*_ ≠ *v*_*h*_, only when nodes *v*_*i*_ and *v*_*h*_ are structurally equivalent [39,40], i.e., nodes *v*_*i*_ and *v*_*h*_ link up exactly the same set of other nodes, $s_{i,j}^{CD}=s_{h,j}^{CD}$. However, structural equivalence is an extremely strict definition and it is unlikely to be met, so CD index can overcome the disadvantage that too many equal similarity values of other indices to some extent. It's worth mentioning that, if *c*_*i*,*j*_ = 0 for *i* = *j* in Eq 2, some elements of node coordinate are futile when calculating CD index. For example, given two node coordinates *v*_1_ = (1,0.2,0.4) and *v*_2_ = (0.2,1,0.5), according to Eq 3, then $s_{1,2}^{CD}=0.4822$. If *c*_*ij*_ = 0 for *i* = *j*, then *v*_1_ = (0,0.2,0.4) and *v*_2_ = (0.2,0,0.5), so $s_{1,2}^{CD}=0.8305$ and the element 0.2 is futile. So we define *c*_*i*,*j*_ = 1 for *i* = *j* in Eq 2.

#### Local Community Density index (LD)

A series of LCP-based (Local-community-paradigm-based) indices were proposed in reference [3], but only number of links between neighbor nodes is considered. Inspired by the idea of reference [3], we universalize LCP as LD

$$s_{i,j}^{LD}=\sum_{p,q\in \Gamma \left(i\right)\cap \Gamma \left(j\right)}s^{*}{}_{p,q}$$ (4)

where s^*^ is a kind of node similarity index. Then we can define *CD*-*LD* index as

$$s_{i,j}^{CD-LD}=\sum_{p,q\in \Gamma \left(i\right)\cap \Gamma \left(j\right)}s^{CD}{}_{p,q}$$ (5)

and *CD* * *LD* as

$$s_{i,j}^{CD*LD}=s_{i,j}^{CD}\cdot s_{i,j}^{CD-LD}$$ (6)

For different threshold value *k*, we can get corresponding CD-k, CD-LD-k and CD*LD-k indices. The threshold *k* of CD, CD-LD and CD*LD is diameter of network.

### Data

In this paper, 10 representative networks from different fields are analyzed: (1) USAir [4]. The USAir transportation network contains 332 airports and 2126 airlines. (2) PB [4]. The pol-blog network is extracted from a set of weblogs about US politics. (3) INT [16]. The router network has 5022 nodes and 6258 links. (4) Neural [22,41]. A network represents the connection of frontal ganglia of nematode worm C. elegans. (5) Word [42]. This is an adjacency network of common adjectives and nouns in the novel David Copperfield by Charles Dickens. (6) NS [42]. In this network, nodes and links represent scientists and coauthor-ships between them respectively. (7) Grid [3]. This is an electrical power grid in western US, nodes representing generators, substations and transformers, edges representing high tension lines between them. (8) FT [43]. American college football team network, collected by Girvan and Newman, contains 115 nodes and 613 edges where nodes represent college football teams and edges represent schedule of competition between teams. (9) Email [44]. This is the giant component of email network which contains 1133 users of University at URV in Tarragona, Spain. (10) Jazz [4]. This is a network of jazz bands, and a link between two bands is established if they had common musician. In this paper, isolated nodes of networks are not considered. Table 1 shows the basic topological features of eleven example networks.

**Table 1:** Basic topological features of example networks. Where, *e* is the efficiency of a network and defined as $e=\frac{2}{n(n-1)}\sum_{i,j\in V,i\neq j}l_{ij}^{-1}$ [51], *c* is clustering coefficient [4], *r* is assortative coefficient [33], *h* is degree heterogeneity and defined as $h=\frac{\langle k^{2}\rangle }{{\langle k\rangle }^{2}}$, where 〈*k*〉 is average degree of a network [16]. *d* is the diameter of a network. *lcp* is the correlation between LCP and CN indices presented in [3]. For more definitions and details of the mentioned topological measures, please reference to [51–53].

	n	m	e	c	r	h	d	lcp
**USAir**	332	2126	0.406	0.749	-0.208	3.46	6	0.9799
**PB**	1224	19090	0.397	0.361	-0.079	3.13	8	0.9286
**INT**	5022	6258	0.167	0.033	-0.138	5.05	15	0.8067
**Neural**	297	2148	0.308	0.2924	-0.1632	1.8008	5	0.9056
**Word**	112	425	0.442	0.1728	-0.1293	1.8149	5	0.8528
**NS**	1461	2742	0.016	0.878	0.462	1.85	17	0.9474
**Grid**	4941	6594	0.063	0.107	0.003	1.45	46	0.8456
**FT**	115	613	0.4504	0.4032	0.1624	1.01	4	0.8931
**Email**	1133	5451	0.2999	0.254	0.0782	1.94	8	0.8538
**Jazz**	198	2742	0.5132	0.633	0.0202	1.3951	6	0.9484

### Methods

To test the accuracy of an algorithm, all existing links, *E*, are divided into two sections randomly: training set *E*^*T*^, as known information, is used for calculating similarity index and contains 90% of *E*, while probe set *E*^*P*^, as unknown information, is used for testing algorithmic accuracy and contains 10% of *E*. Clearly, *E* = *E*^*T*^∪*E*^*P*^ and *E*^*T*^∩*E*^*P*^ = ∅.

Two main metrics, which emphasize different aspects, can be used to evaluate the performance of link prediction algorithms: AUC (area under the receiver operating characteristic curve) [45, 46] and Precision [6, 47, 48]. According to the analysis and comparison in reference [47], AUC value can be deceptive and Precision is more suitable choice for evaluating the performance of link prediction algorithms. So the Precision value is considered in this paper.

For calculating Precision values [47, 48], all the nonexistent links need to be ranked in decreasing order according to their scores. Then in top-*L* links, if there are *l* links successfully predicted, then

$$Precision=\frac{l}{L}$$ (7)

where *L* is the number of links in *E*∩*E*^*P*^. Clearly, higher precision values mean higher prediction accuracy of index.

To ensure that the comparison is fair, in this paper, all Precision values in Table 2 are the average values computed on 100 iterations. For each iteration, a set of 90% randomly selected network interactions was used as training set for the algorithms and the remaining 10% interactions were used for the test set.

**Table 2:** Precision values of link prediction indices on example networks. The order of the networks is organized according to their increasing assortative coefficient (from negative to positive), and values in brackets under the network names are the coefficient of each network.

	1	2	3	4	5	6	7	8	9	10
	USAir	Neural	INT	Word	PB	Grid	Jazz	E-mail	FT	NS
	(-0.208)	(-0.1632)	(-0.138)	(-0.1293)	(-0.079)	(0.003)	(0.0202)	(0.0782)	(0.1624)	(0.462)
**CN**	0.3862	0.0943	0.0566	0.0744	0.1764	0.0568	0.5062	0.1421	0.2894	0.5098
**Salton**	0.0554	0.0233	0	0	0.0099	0.0152	0.5404	0.044	0.3484	0.5913
**PA**	0.3313	0.0579	0.0192	0.1023	0.0671	0.0008	0.1305	0.0173	0.0003	0.04
**Sorensen**	0.0742	0.0288	0	0	0.0196	0.0147	0.5287	0.0648	0.3452	0.5905
**LHN**	0.0103	0	0	0	0.0005	0.0121	0.1018	0.0018	**0.3903**	0.2429
**RA**	**0.4712**	0.0994	0.0195	0.0614	0.1562	0.0326	0.5397	0.1447	0.2826	**0.7454**
**LP-3**	0.3822	0.0993	0.0577	0.0812	**0.1828**	0.0614	0.4727	0.1368	0.2631	0.4982
**LP-4**	0.3728	0.0958	0.0511	**0.107**	0.1821	**0.0648**	0.448	0.133	0.271	0.4982
**LRW**	0.1191	**0.1415**	0.0397	0.067	0.095	0.0094	0.3569	0.0667	0.2531	0.3927
**LB**	0.3697	0.0862	**0.207**	0.1019	0.1462	0.0579	0.2753	0.0852	0.1865	0.3655
**CAR**	0.3771	0.0921	0.062	0.0549	0.1729	0.035	0.5187	0.1402	0.3366	0.5062
**CPA**	0.3778	0.098	0.0208	0.0695	0.173	0.005	0.5168	0.1293	0.3323	0.3193
**CAA**	0.3792	0.1001	0.0617	0.047	0.1731	0.0314	0.5338	0.1434	0.3576	0.584
**CRA**	0.4028	0.115	0.0617	0.0491	0.1796	0.0314	**0.5592**	**0.1567**	0.3581	0.6269
**CJC**	0.3587	0.0701	0.0607	0.0249	0.1616	0.0314	0.5568	0.1523	0.3563	0.5585
**CD-LD**	0.3841	0.0977	0.0515	0.0298	0.1765	0.0562	0.5128	0.1478	0.3111	0.5331
**CD-LD-2**	0.4178	0.0837	0.0655	0.106	0.1758	0.0621	0.5164	0.1381	0.3387	0.54
**CD-LD-3**	0.4178	0.0837	0.0687	0.1023	0.1764	0.0621	0.5236	0.1344	0.3548	0.5436
**CD-LD-4**	0.4178	0.0977	0.0703	0.098	0.1758	0.0621	0.5236	0.141	0.3468	0.5418
**CD-LD-5**	0.4178	0.0977	0.0687	0.0977	0.1755	0.0621	0.5236	0.1447	0.3468	0.5418
**CD-LD-6**	0.4178	0.0977	0.0703	0.0977	0.1755	0.0621	0.5236	0.1458	0.3468	0.54
CD*LD	0.3839	0.0915	0.0475	0.0395	0.1768	0.0562	0.5186	0.1465	0.3192	0.5076
CD*LD-2	0.4131	0.0814	0.0495	0.0447	0.1755	0.0621	0.5236	0.1341	0.3306	0.54
CD*LD-3	0.4178	0.0744	0.0607	0.0726	0.1767	0.0621	0.5345	0.1337	0.3629	0.54
CD*LD-4	0.4178	0.0837	0.0527	0.0726	0.1764	0.0621	0.5345	0.141	0.3629	0.5327
CD*LD-5	0.4178	0.0837	0.0591	0.0726	0.1764	0.0621	0.5345	0.1443	0.3629	0.5255
CD*LD-6	0.4178	0.0837	0.0607	0.0781	0.1767	0.0621	0.5345	0.1454	0.3629	0.5236
**CD**	0.0085	0.0051	0.0002	0.0058	0.001	0.0106	0.2589	0.012	0.3477	0.12
**CD-2**	0.0188	0.0093	0	0.0078	0.003	0.0152	0.36	0.0308	0.2419	0.3945
**CD-3**	0.0188	0.0047	0	0.0078	0.0015	0.0136	0.36	0.0484	0.371	0.2709
**CD-4**	0.0141	0.0047	0	0.0078	0.0012	0.0152	0.3055	0.0319	0.379	0.2073
**CD-5**	0.0141	0.0047	0	0.0078	0.0018	0.0121	0.2945	0.0117	0.379	0.1855
**CD-6**	0.0141	0.0047	0	0.0078	0.0018	0.0106	0.2945	0.0099	0.379	0.1527
**CDI**	0.3352	0.0558	0.0272	0.0744	0.0726	0.0083	0.1404	0.0257	0.0032	0.0938
**RP**	0.0047	0.0047	0	0.0065	0.0025	0	0.0161	0.0018	0.0102	0

In this paper, prediction accuracies of fifteen existing similarity indices including Common Neighbors Index (CN) [21,49], Salton index (Salton) [14], Sorensen Index (Sorensen) [5], Leicht-Holme-Newman Index (LHN) [15], Local Path Index(LP) [20], Local Random Walk (LRW) [21,41], LB(local blocking) index [50], Preferential Attachment (PA) [29], Resource Allocation Index (RA) [16], and LCP-based indices[3] were compared with the proposed CD-based similarity indices. CN is based on the hypothesis that two nodes are more likely to generate a link in the near future if they have more common neighbors. On networks with high clustering coefficient, CN can provide competitively accurate prediction results compared with other indices [22]. Salton, Sorensen and LHN are expanded from CN and they are neighbor-based methods. LP is a popular distance-based method and it can give more accurate predictions than other distance-based methods such as Katz and LHN-II on some networks with small average shortest distance [6]. LP-3 and LP-4 represent *S*^*LP*^ = *A*^2^ + *εA*^3^ and *S*^*LP*^ = *A*^2^ + *εA*^3^ + *ε*^2^*A*^4^ respectively. LRW is a famous index based on local random walk[21,41]. LB index is based on the idea of local blocking and link density between them [50]. PA is the well-known preferential attachment mechanism proposed by BARABASI A-L to generate scale-free networks. RA is a high-precise and low-complexity similarity index based on resource allocation process. LCP-based indices including CAR, CPA, CAA, CRA and CJC are a series of marvelous indices based on local communities.

For LP index, *ε* is a free parameter and *ε* = 0.01 in this paper. For LRW index, the step of random walk is set as 3. Table 3 shows affiliations of CD-based, CD-k, CD-LD-k, CD*LD-k and CDI indices, where *k* is the threshold in Eq 1. In addition, the performance of a random predictor was also compared with the proposed similarity indices and the random predictor is computed considering a ranking obtained as the random permutation of the 10% test-links [3].

**Table 3:** Affiliations of the proposed indices. CD-based indices represent four proposed indices as CD-LD-k, CD*LD-k, CD-k and CDI, where *k* is the value of threshold in Eq 1.

CD-based			
CD-LD-k	CD*LD-k	CD-k	CDI

## Results and Discussion

The proposed CD-based algorithms and fifteen existing methods were compared on ten real networks, and their Precision values are shown in Table 2 and the best value of each network is emphasized by boldface. The definitions of fifteen existing methods are introduced in Methods section and the basic topological features of ten example networks are shown in Table 1.

We can clearly see from Table 2, CD-based indices can improve prediction accuracy of some existing indices such as CN, Salton, PA and Sorensen on all experimental networks, LHN on all networks except FT, RA on INT, Word, PB, Grid, E-mail and FT, LP-k on USAir, INT, Jazz, E-mail, FT and NS, LRW on all networks except Neural, LB on all networks except INT, all LCP-based indices on USAir, INT, Word, Grid and FT.

From another point of view, RA performs best on networks USAir and NS with Precision values as 0.4712 and 0.7454 respectively, and the performance of CD-based indices takes second place on network USAir with Precision value as 0.4178. On network Neural, CD-based indices can obtain greater Precision values than other indices except LRW and LCP-based indices. On network INT, the Precision value of CD-based indices is greater than values of other indices while less than the greatest value 0.207 of LB index.

LP-k indices perform best on networks Word, PB and Grid, and CD-based indices take second place on networks Word and Grid. LCP-based indices perform best on network Jazz with Precision value as 0.5592 and on network E-mail with 0.1567, while CD-based indices have 0.1478 on network E-mail which is less than that of LCP-based indices. On network FT, LHN index performs best and CD-based indices perform better than LCP-based indices satisfactorily. On the whole, CD-based and LCP-based always perform well on all networks although CD-based indices have not the greatest Precision values. Therefore, CD-based and LCP-based indices are more robust than other indices.

For Precision values in Table 2, all existing indices except PA, LP-k and LB obtain prediction accuracies less than 0.1 on networks INT, Grid and Word with low clustering coefficient, even Salton, Sorensen and LHN get 0 on INT and Word. But these indices can obtain satisfactory prediction accuracy on high clustering coefficient networks FT, Jazz, and NS. As a matter of fact, most of existing indices which are considered in this paper as well as CD-LD-k and CD*LD-k indices are based on local information of networks, so their prediction accuracy will be inevitably influenced by clustering coefficient and these indices perform better on networks with high clustering coefficient. While we can find from Table 2 that CD-LD-k and CD*LD-k indices can improve prediction accuracy of existing indices on networks with low clustering coefficient on the whole.

In addition, relative precision of each method on each network is utilized to discuss average performance and robustness of link prediction indices. The relative precision is computed as the precision of a method on a network divided by the precision of the random predictor on the same network and whose precision values on each network are shown in the bottom line of Table 2. The relative precision of each index on each network is calculated and shown in Table 4. Mean and minimum relative precision values of each index are also calculated and mean value is used as an indicator of average performance, whereas the minimum value is used as an indicator of robustness. From Table 4, CRA index of LCP-based indices has the best average performance and LP4 has the best robustness. On the whole, the average performance and robustness of CD-LD-k and CD*LD-k indices are satisfactory with mean relative precision values more than 45 and minimum relative precision values more than 10. As a matter of fact, from a statistical point, CN, RA, LP-k, LB, LCP-based, CD-LD-k and CD*LD-k indices provide a very similar performance.

**Table 4:** Relative precision of each index. The order of the networks is organized according to their increasing assortative coefficient (from negative to positive), and values in brackets under the network names are the coefficient of each network. Mean and minimum relative precision values of each index are shown in last two columns. The mean value is used as an indicator of average performance and minimum value is used as a measure of robustness performance.

	1	2	3	4	5	6	7	Mean	Minimum
	USAir	Neural	Word	PB	Jazz	Email	FT		
	(-0.208)	(-0.1632)	(-0.1293)	(-0.079)	(0.0202)	(0.0782)	(0.1624)		
**CN**	82.26059	20.2745	11.44615	70.56	31.44099	77.58659	28.37255	45.99163	11.44615
**Salton**	11.8002	5.0095	0	3.96	33.56522	24.024	34.15686	16.07368	0
**PA**	70.5669	12.4485	15.73846	26.84	8.10559	9.445799	0.029412	20.45352	0.029412
**Sorensen**	15.8046	6.192	0	7.84	32.83851	35.3808	33.84314	18.84272	0
**LHN**	2.1939	0	0	0.2	6.322981	0.9828	**38.26471**	6.852055	0
**RA**	**100.3656**	21.371	9.446154	62.48	33.52174	79.00619	27.70588	47.69951	9.446154
**LP3**	81.40859	21.3495	12.49231	**73.12**	29.36025	74.69279	25.79412	45.45965	12.49231
**LP4**	79.40639	20.597	**16.46154**	72.84	27.82609	72.61799	26.56863	45.18823	**16.46154**
**LRW**	25.3683	**30.4225**	10.30769	38	22.1677	36.4182	24.81373	26.78544	10.30769
**LB**	78.65957	18.34043	15.67692	58.48	17.09938	47.33333	18.28431	36.2677	15.67692
**CAR**	80.32229	19.8015	8.446154	69.16	32.21739	76.54919	33	45.64236	8.446154
**CPA**	80.47139	21.07	10.69231	69.2	32.09938	70.59779	32.57843	45.24419	10.69231
**CAA**	80.76959	21.5215	7.230769	69.24	33.15528	78.29639	35.05882	46.46748	7.230769
**CRA**	85.79639	24.725	7.553846	71.84	**34.73292**	**85.55819**	35.10784	**49.3306**	7.553846
**CJC**	76.40309	15.0715	3.830769	64.64	34.58385	83.15579	34.93137	44.65948	3.830769
**CD-LD**	81.81329	21.0055	4.584615	70.6	31.85093	80.69879	30.5	45.86473	4.584615
**CD-LD-2**	88.99139	17.9955	16.30769	70.32	32.07453	75.40259	33.20588	47.7568	16.30769
**CD-LD-3**	88.99139	17.9955	15.73846	70.56	32.52174	73.38239	34.78431	47.71054	15.73846
**CD-LD-4**	88.99139	21.0055	15.07692	70.32	32.52174	76.98599	34	48.41451	15.07692
**CD-LD-5**	88.99139	21.0055	15.03077	70.2	32.52174	79.00619	34	48.67937	15.03077
**CD-LD-6**	88.99139	21.0055	15.03077	70.2	32.52174	79.60679	34	48.76517	15.03077
CD*LD	81.77069	19.6725	6.076923	70.72	32.21118	79.98899	31.29412	45.96206	6.076923
CD*LD-2	87.99029	17.501	6.876923	70.2	32.52174	73.21859	32.41176	45.81719	6.876923
CD*LD-3	88.99139	15.996	11.16923	70.68	33.19876	73.00019	35.57843	46.94486	11.16923
CD*LD-4	88.99139	17.9955	11.16923	70.56	33.19876	76.98599	35.57843	47.78276	11.16923
CD*LD-5	88.99139	17.9955	11.16923	70.56	33.19876	78.78779	35.57843	48.04016	11.16923
CD*LD-6	88.99139	17.9955	12.01538	70.68	33.19876	79.38839	35.57843	48.26398	12.01538
**CD**	1.8105	1.0965	0.892308	0.4	16.08075	6.551999	34.08824	8.702898	0.4
**CD-2**	4.0044	1.9995	1.2	1.2	22.36025	16.8168	23.71569	10.18523	1.2
**CD-3**	4.0044	1.0105	1.2	0.6	22.36025	26.4264	36.37255	13.13916	0.6
**CD-4**	3.0033	1.0105	1.2	0.48	18.97516	17.4174	37.15686	11.32046	0.48
**CD-5**	3.0033	1.0105	1.2	0.72	18.29193	6.388199	37.15686	9.681541	0.72
**CD-6**	3.0033	1.0105	1.2	0.72	18.29193	5.4054	37.15686	9.541141	0.72
**CDI**	71.3976	11.997	11.44615	29.04	8.720497	14.0322	0.313725	20.99245	0.313725

Considering global networks information, CD may get poor prediction accuracy on relatively higher clustering coefficient networks. So threshold *k* in Eq 1 is set smaller as 2, 3, 4, 5 and 6 according to vast experiments, and their corresponding CD indices are denoted as CD-2, CD-3, CD-4, CD-5, CD-6 in Tables 2 and 4. Compared with other indices in Table 2, CD and CD-k indices perform far from satisfactory. Moreover, we find out that the performance of CD and CD-4 indices on networks Neural, INT, Word, and PB with negative assortative coefficient is worse than the performance on networks Grid, Jazz, Email, FT and NS with positive assortative coefficient. In spite of Precision values of CD-2 and CD-3 on negative assortative network USAir are greater than the values on positive assortative network Grid, the performance of CD and CD-k indices on network USAir is worse than on positive assortative networks Jazz, FT and NS. From Table 4 on Relative precision, more clearly, the performance of CD and CD-k indices on negative assortative networks USAir, Neural, Word and PB is worse than on positive assortative networks Jazz, Email and FT. On the whole, CD and CD-k indices are more appropriate for link prediction on positive assortative networks.

On the contrary, from Table 2, prediction accuracies of PA index on negative assortative networks USAir, Neural, INT, Word and PB are greater than those on positive assortative networks Grid, Email, FT and NS. From Table 4, more obviously, the Relative precision values of PA index on negative assortative networks USAir, Neural, Word and PB are greater than those on positive assortative networks Jazz, Email and FT. Furthermore, its mean value of Precision values on all networks with negative assortative coefficient is 0.1156 which is much higher than such value on all positive assortative coefficient networks as 0.0378. So we think PA index is suitable for networks with negative assortative coefficient and it is different from the idea in reference [6] that the poor performance of PA index on Grid network is caused by influence of rare long geographical distance.

### Improvement

According to the discussion in above section and results of CD and CD-k indices, a conclusion was reached that the proposed CD and CD-k indices perform better on networks with positive assortative coefficient than on networks with negative assortative coefficient. For the networks with negative assortative coefficient, improvements of CD and CD-k indices are based on the following consideration. In reference [33], for a network, Newman thought that the assortative coefficient is positive or negative corresponding to assortative or disassortative mixing respectively. Assortative mixing means that high degree nodes tend to connect with high degree nodes and vice versa. Disassortative mixing means that high degree nodes tend to connect with low degree nodes and vice versa. Newman applied assortative coefficient to the network model of Barabasi and Albert (BA) [29] and found out that these kinds of network are disassortative mixing. In other words, the assortative coefficient of BA network is negative. Because an evolving network model corresponds to a link prediction method [16], an algorithm can perform better on the networks corresponding to its evolutionary mechanism. As we can see from Table 2, PA index, as the preferential attachment mechanism, performs better on most negative assortative networks compared with its performance on positive assortative networks. So we think that the network growth by CD or CD-k mechanism will have positive assortative coefficient. In order to improve CD and CD-k indices on negative assortative networks, the advantages of CD and PA indices are taken into account and improvement denoted as CDI is made as follows

$$s_{ij}^{CDI}=\frac{\left(C_{i},C_{j}\right)}{\left\|C_{i}\right\|\cdot \left\|C_{j}\right\|}\times \left(k_{i}\times k_{j}\right)$$ (8)

Improved index CDI was also applied to the ten real networks, and its Precision values and Relative precision values are shown in Table 2 and Table 4 respectively. Apparently, from Table 2, on networks USAir, Neural, INT, Word and PB with negative assortative coefficient, Precision values obtained by CDI have been improved compared with CD and CD-k indices. However, on networks Grid, Jazz, FT and NS with positive assortative coefficient, Precision values obtained by CDI are reduced compared with CD and CD-k indices. In brief, CDI index can improve prediction accuracy of CD and CD-k indices on negative assortative coefficient networks. From Table 2, we can also draw a conclusion that CDI index can improve prediction accuracy of PA index both on negative and positive assortative networks except networks Neural and Word. What’s more, from Table 4, CDI has greater average performance and robustness than PA index.

In addition, the complexity of the proposed link prediction method is O(*n*^2^) at most. In Eq 1, all the shortest paths between nodes in a network should be calculated. But the traditional shortest path algorithms are time-consuming such as Dijkstra and Floyd with complexity as high as O(*n*^3^). For improving algorithm complexity, we applied breadth-first search to network adjacent matrix and proposed a new method to calculate all the shortest paths between nodes in un-weighted and undirected connected networks with time complexity as O(*n*^2^) at most. The proposed shortest path algorithm is described as follows:

**Input**: adjacency matrix *A* of an un-weighted and undirected connected network *G* = (*V*, *E*).

**Output**: distance matrix *D* of *G*.

**Step 1:** Set integer variables *k* = 1, *i* = 1 and distance matrix *D* = *A*.

**Step 2:** Find coordinates of element *k* in *i*th row of *D* as $V_{k}^{i}=\left(v_{k}^{1},v_{k}^{2},v_{k}^{3},\cdots \right)$.

**Step 3:** Find coordinate of element 1 in $v_{k}^{j}$th row of *A* as $U_{k}^{i,j}$, where vkj∈Vki.

**Step 4:** Set $U_{k}^{i}=U_{k}^{i,1}\cap U_{k}^{i,2}\cap U_{k}^{i,3}\cap \cdots =\left(u_{k}^{i,1},u_{k}^{i,2},u_{k}^{i,3},\cdots ,u_{k}^{i,y}\right)$.

**Step 5:** Set $u_{k}^{i,x}$th element as *k* + 1 in *i*th row of *D*, where $u_{k}^{i,x}\in U_{k}^{i}$.

**Step 6:** Increase *k* as *k* = *k* + 1 and repeat from Step 2 to Step 5 until $V_{k}^{i}$ is empty.

**Step 7:** Increase *i* as *i* = *i* + 1, let *k* = 1 and repeat from Step 2 to Step 6 until *i* = *n*.

Because calculating CD index in Eq 3 needs complexity at most O(*n*^2^) and calculating LD index in Eq 4 needs complexity at most O(*n*^3^), so the complexity of CD, CD-k and CDI are O(*n*^2^) and the complexity of CD-LD-k and CD*LD-k are O(*n*^3^). Table 5 shows time complexities of link prediction algorithms.

**Table 5:** Index complexity. All complexities of indices in this table are estimated by the worst condition of networks. CP represents the complexity of an index.

Index	CN	Salton	PA	Sorensen	LHN	RA	LP	LRW	LB	LCP-based	CD-LD-k	CD*LD-k	CD-k	CDI
**CP**	*o*(*n*^2^)	*o*(*n*^2^)	*o*(*n*^2^)	*o*(*n*^2^)	*o*(*n*^2^)	*o*(*n*^3^)	*o*(*n*^3^)	*o*(*n*^3^)	*o*(*n*^2^)	*o*(*n*^3^)	*o*(*n*^3^)	*o*(*n*^3^)	*o*(*n*^2^)	*o*(*n*^2^)

## Conclusions

In this paper, a series of new CD-based indices based on cosine similarity for link prediction are proposed. Fifteen existing similarity indices are compared with the proposed indices and experimental results demonstrate the effectiveness of CD-based indices. Through detailed analysis and comparison according to Precision values, four important points were found out: (i) In spite of influenced by clustering coefficient, the Precision values of CD-LD-k and CD*LD-k indices are obviously superior to the values of some other indices. (ii) CD-LD-k and CD*LD-k indices are robust and they have satisfactory average performance and robustness. (iii) CD and CD-k indices are more appropriate for link prediction on positive assortative networks. (iv) CDI performs better on negative assortative coefficient networks and can improve prediction accuracy of PA index.

Further investigation and improvements will focus on the following aspects. Firstly, the conclusion that CD is suitable for positive assortative coefficient networks and CDI is suitable for negative assortative coefficient networks needs more practical verification. Secondly, the influence of other topological features such as network efficiency and degree heterogeneity on link prediction accuracy should be studied in depth. Thirdly, abundant information can be extracted from *k*-distance matrix *L***,** such as connections between its eigenvectors and network topological features. Fourthly, according to the conclusion that CD is suitable for positive assortative networks and CDI is suitable for negative assortative networks, we guess that evolving networks of CD and CDI should be assortative mixing and disassortative mixing respectively. Fifthly, LD index can be expanded using different similarity indices to calculate the local density. Sixthly, a theoretical strategy is required to tune the threshold *k* in Eq 1. Finally, we hope the link prediction methods presented here can be expanded to other types of networks, such as weighted network, directed network, bipartite network and dynamic network. We hope such improvements and more applications of CD-based methods in the future.
